# Supplementary figures and images for: Molecular evolution of CO2-sensing ab1C neurons underlies divergent sensory responses in the Drosophila suzukii species group
Source: PLoS Genet. 2026 Jan 22;22(1):e1012024. doi: 10.1371/journal.pgen.1012024 (PMC12854461; doi:10.1371/journal.pgen.1012024)

**A**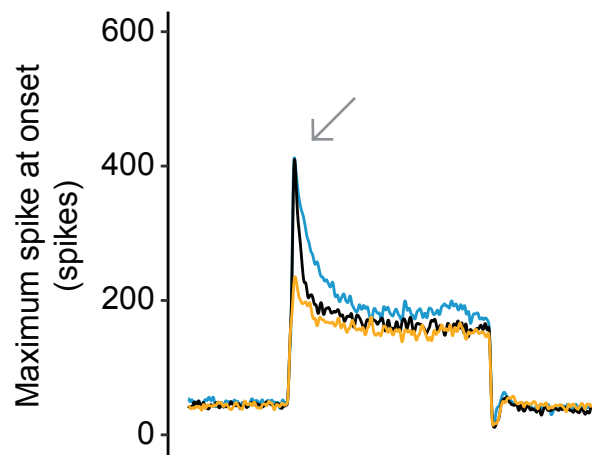**B**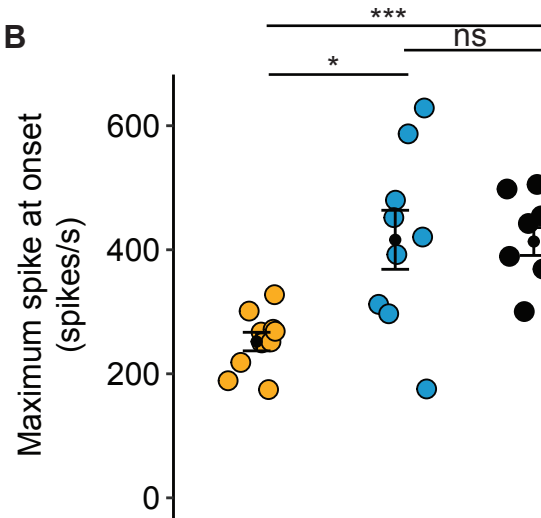**C**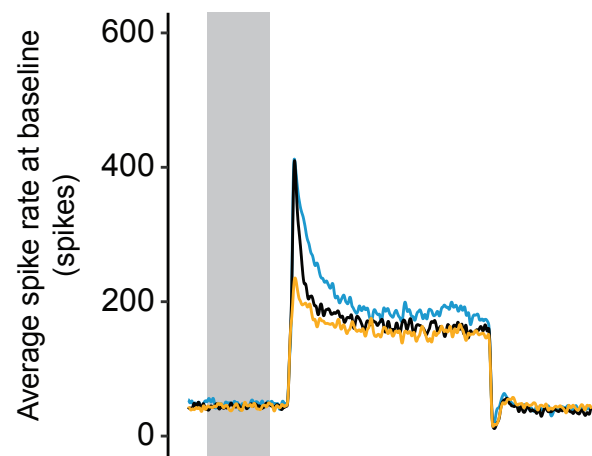**D**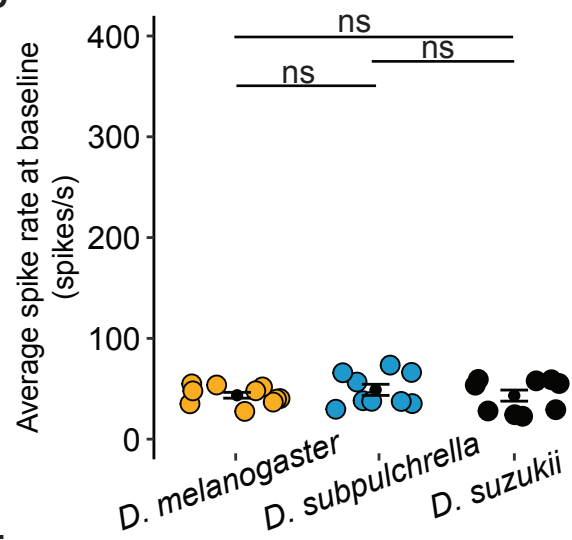**E**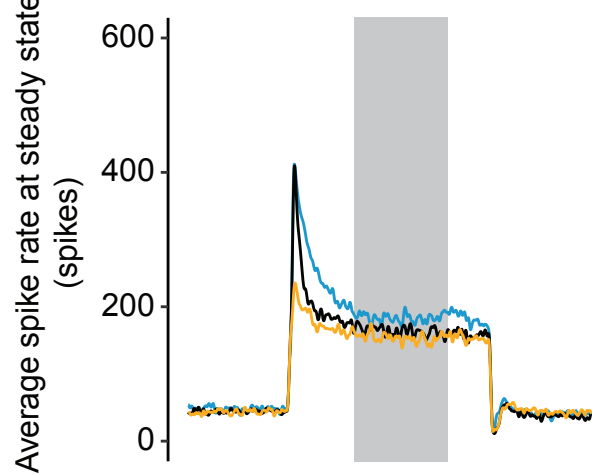**F**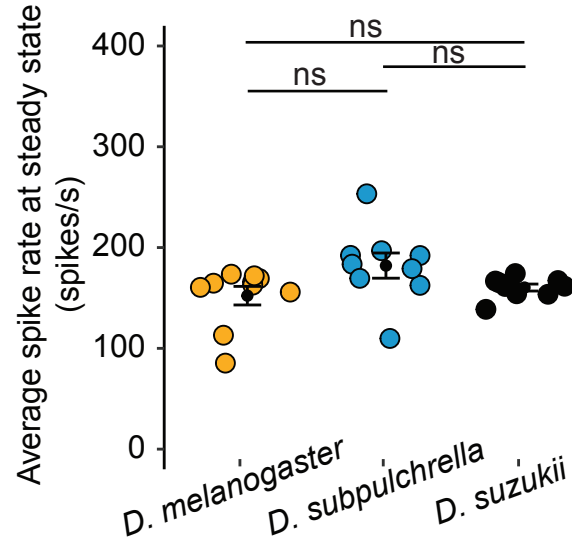

Supplement: S1 Fig — A-B) The maximum spike rate across each ab1c neuron recording is significantly higher in D. suzukii and D. subpulchrella compared to D. melanogaster (Kruskal-Wallis test followed by pairwise Wilcoxon signed-rank test with Holm correction, Q< 0.001, Q = 0.017). C-D) The ab1c spike rate of the three species is not significantly different at baseline (Kruskal-Wallis test followed by pairwise Wilcoxon signed-rank test with Holm correction, Dsub-Dmel: Q = 1.00, Dsuz-Dmel: Q = 1.00, Dsub-Dsuz: Q = 0.89). E-F) The ab1c spike rate of the three species is not significantly different at steady state, which was calculated across 1000 ms (Kruskal-Wallis test followed by pairwise Wilcoxon signed-rank test with Holm correction, Dsub-Dmel: Q = 0.066, Dsuz-Dmel: Q = 1.00, Dsub-Dsuz: Q = 0.056). (PDF) [file pgen.1012024.s001.pdf]

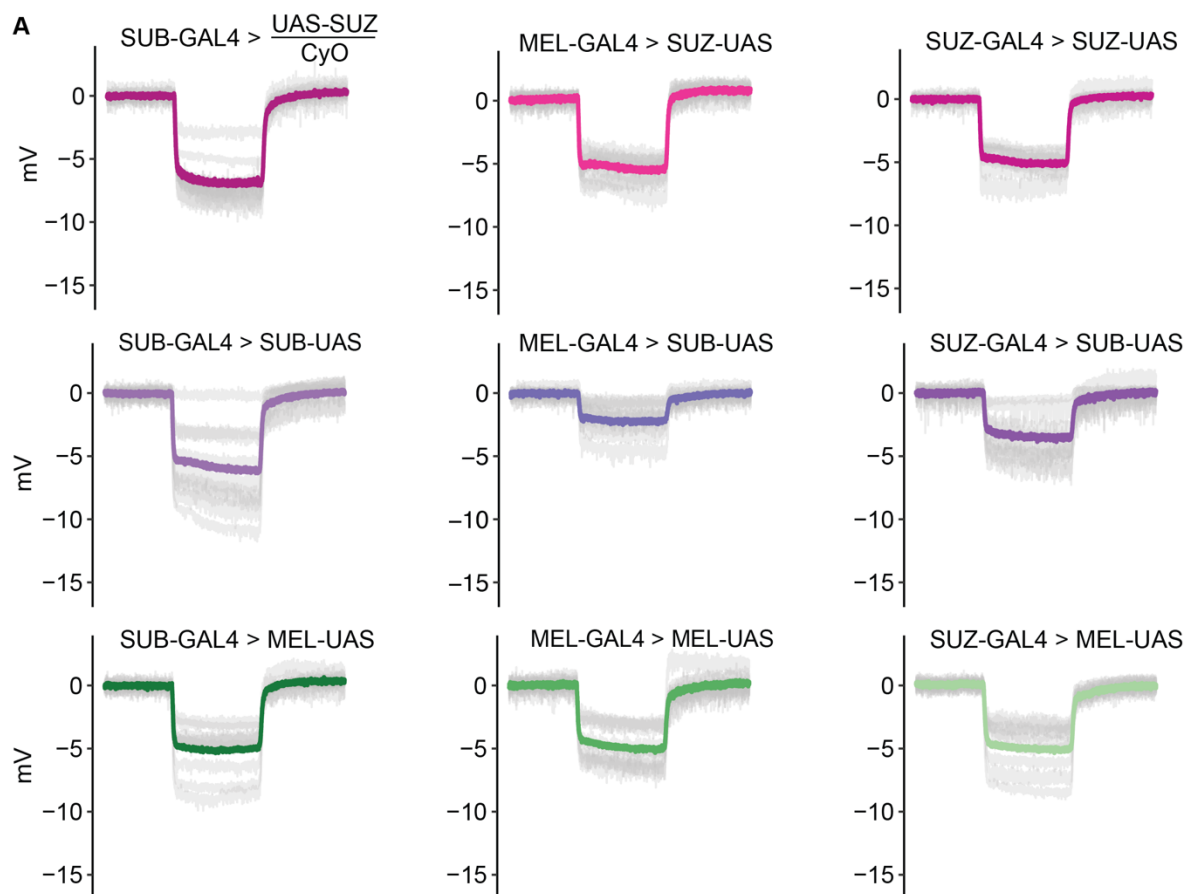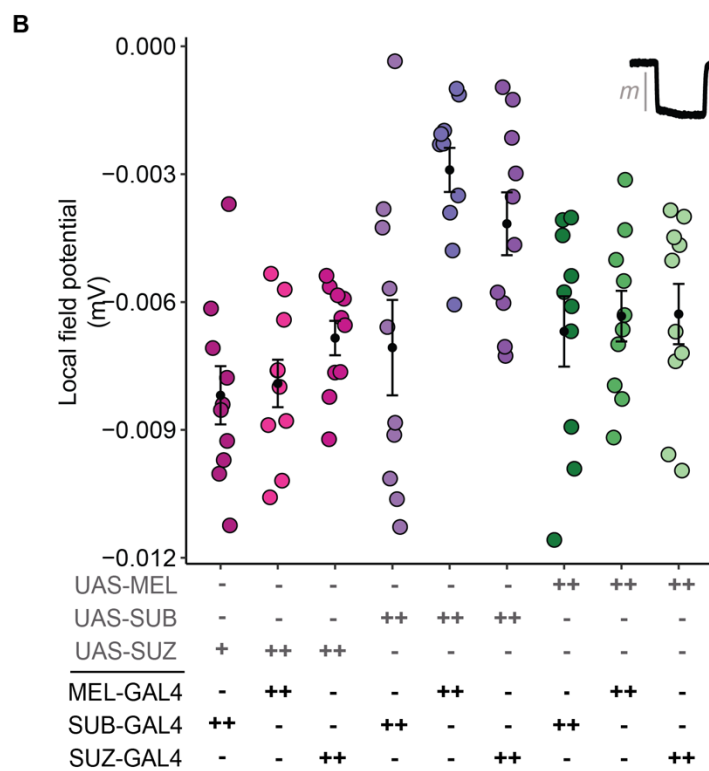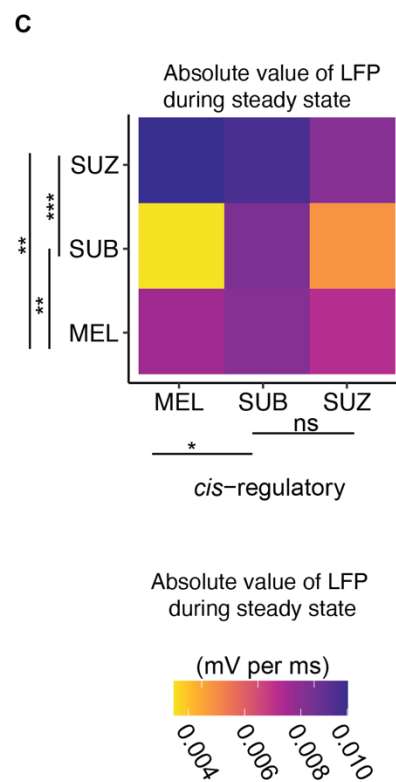

Supplement: S2 Fig — A) LFP recordings for each transgenic line of the ab1c neurons recorded in Fig 4. Each grey line is the average of five replicates from one ab1c neuron, and the colored lines are the average across ten neurons. B) Without correcting for the heterozygous individual, the slope of the LFP at CO2 onset was calculated across 750 ms. For the CDS, transgenic lines with D. suzukii CDS had the highest rate of change, followed by D. melanogaster and then D. subpulchrella (Kruskal-Wallis test followed by pairwise Wilcoxon signed-rank test with Holm correction, Dsub-Dsuz: Q =0.00027, Dsuz-Dmel: Q= 0.01990, Dsub-Dmel: Q =0.01836). For the cis-regulatory element, the D. subpuclrehlla had the highest rate of change and trended lower than the other species elements (Kruskal-Wallis test followed by pairwise Wilcoxon signed-rank test with Holm correction, Dsub-Dsuz: Q= 0.092, Dsuz-Dmel: Q= 0.982, Dsub-Dmel: Q =0.092). (PDF) [file pgen.1012024.s002.pdf]

**A**

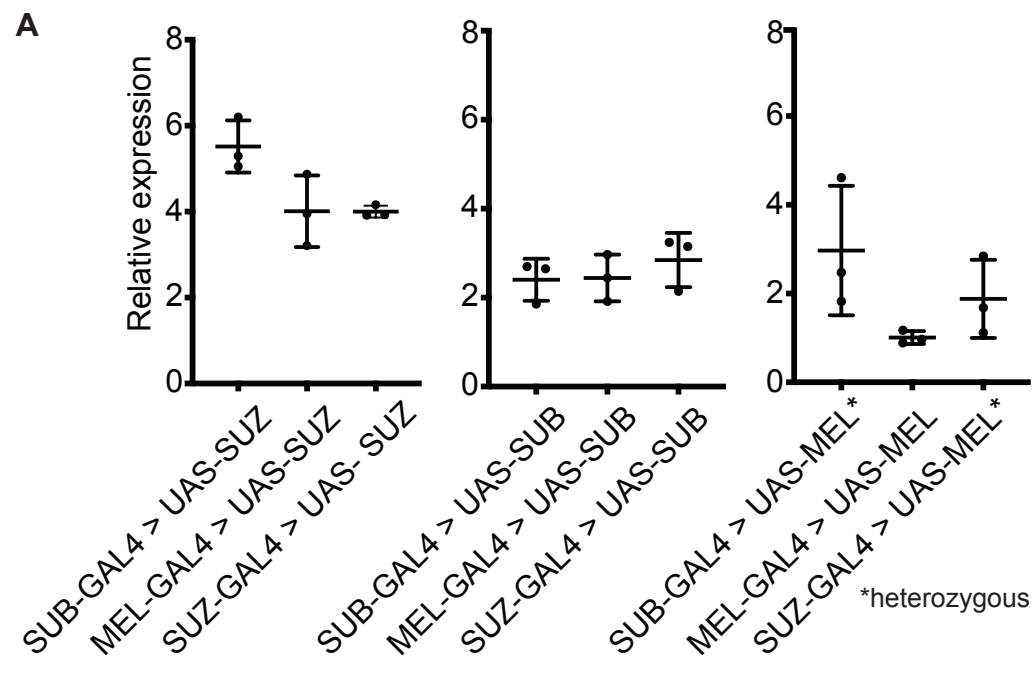

**B**

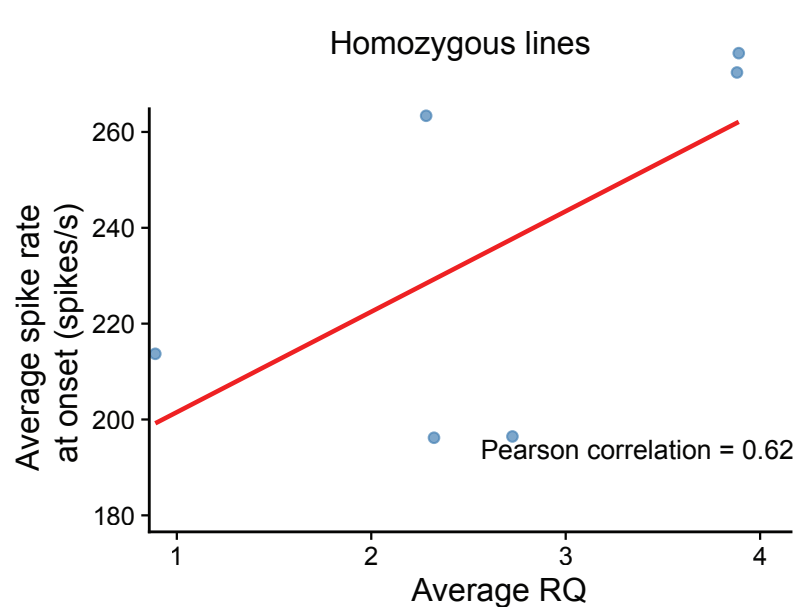

Supplement: S4 Fig — A) qRT-PCR of the transgenic lines, normalized to the line containing homozygote MEL-GAL4 and UAS-MEL. Note that we were not able to obtain homozygous flies for two strains at the time of the experiment. B) Correlation between average spike rate and expression levels for the six lines that have homozygous flies for both quantitative RT-PCR and SSE recordings. (PDF) [file pgen.1012024.s004.pdf]
