# Supplementary material for: Molecular evolution of CO2-sensing ab1C neurons underlies divergent sensory responses in the Drosophila suzukii species group
Source: PLoS Genet. 2026 Jan 22;22(1):e1012024. doi: 10.1371/journal.pgen.1012024 (PMC12854461; doi:10.1371/journal.pgen.1012024)
Supplement: S3 Fig — A) Comparison of Gr63a coding sequences among D. suzukii, D. subpulchrella, and D. melanogaster. B) Predicted spike rates at CO2 onset for the SUB-GAL4 > UAS-SUZ individual as well as the SUB-GAL4 > UAS-SUB control. The predicted spike rate of SUB-GAL4 > SUB-UAS and the actual spike rate are very similar (Kruskal-Wallis test followed by pairwise Wilcoxon signed-rank test with Holm correction, Q = 0.19, mean of B = 224, mean of estimated B = 228), which gave us confidence that the predicted homozygous spike rate for SUB-GAL4 > UAS-SUZ is robust. C) Heat map comparing the spike rate between homozygous transgenic lines, including the modeled SUB-GAL4 > UAS-SUZ value, carrying subpulchrella and melanogaster proteins with cis-regulatory sequences from the three species. The D. suzukii CDS (Kruskal-Wallis test followed by pairwise Wilcoxon signed-rank test with Holm correction, Dmel–Dsuz: Q = 1.1e-08 and Dsub-Dsuz: Q = 6.3e-07) and D. subpulchrella cis-regulatory element (Kruskal-Wallis test followed by pairwise Wilcoxon signed-rank test with Holm correction, Q = 0.0139 between D. melanogaster and D. subpulchrella and Q = 0.004 between D. subpulchrella and D. suzukii) resulted in the highest spike rate. D) Spike rate comparisons of homozygote transgenic lines carrying subpulchrella and suzukii proteins with regulatory sequences from the three species. E) Spike rate comparisons of homozygote transgenic lines carrying melanogaster and suzukii regulatory sequences with CDS from the three species. (PDF) [file pgen.1012024.s003.pdf]

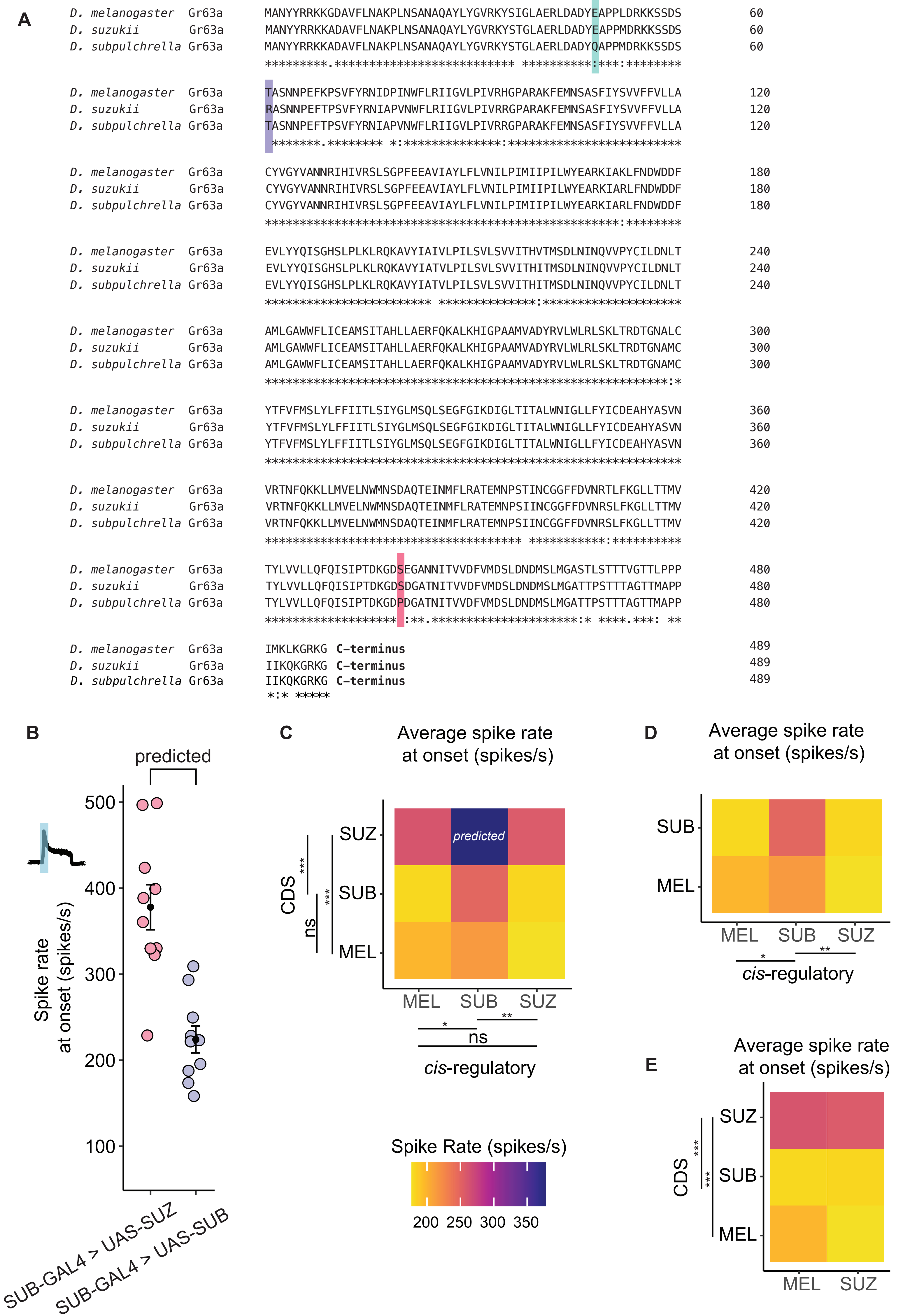
A
D. melanogaster Gr63a MANYYRRKKGDAVFLNAKPLNSANAQAYLYGVRKYSIGLAERLDADYEAPPLDRKKSSDS 60
D. suzukii Gr63a MANYYRRKKADAVFLNAKPLNSANAQAYLYGVRKYSTGLAERLDADYEAPPMDRKKSSDS 60
D. subpulchrella Gr63a MANYYRRKKADAVFLNAKPLNSANAQAYLYGVRKYSTGLAERLDADYQAPPMDRKKSSDS 60
D. melanogaster Gr63a TASNNPEFKPSVFYRNIDPINWFLRIIGVLPIVRHGPARAKFEMNSASFIYSVVFFVLLA 120
D. suzukii Gr63a RASNNPEFTPSVFYRNIAPVNWFLRIIGVLPIVRRGPARAKFEMNSASFIYSVVFFVLLA 120
D. subpulchrella Gr63a TASNNPEFTPSVFYRNIAPVNWFLRIIGVLPIVRRGPARAKFEMNSASFIYSVVFFVLLA 120
D. melanogaster Gr63a CYVGYVANNRIHIVRSLSGPFEEAVIAYLFLVNILPIMIIPILWYEARKIAKLFNDWDDF 180
D. suzukii Gr63a CYVGYVANNRIHIVRSLSGPFEEAVIAYLFLVNILPIMIIPILWYEARKIARLFNDWDDF 180
D. subpulchrella Gr63a CYVGYVANNRIHIVRSLSGPFEEAVIAYLFLVNILPIMIIPILWYEARKIARLFNDWDDF 180
D. melanogaster Gr63a EVLYYQISGHSLPLKLRQKAVYIAIVLPILSVLSVVITHVTMSDLNINQVVPYCILDNLT 240
D. suzukii Gr63a EVLYYQISGHSLPLKLRQKAVYIATVLPILSVLSVVITHITMSDLNINQVVPYCILDNLT 240
D. subpulchrella Gr63a EVLYYQISGHSLPLKLRQKAVYIATVLPILSVLSVVITHITMSDLNINQVVPYCILDNLT 240
D. melanogaster Gr63a AMLGAWWFLICEAMSITAHLLAERFQKALKHIGPAAMVADYRVLWLRLSKLTRDTGNALC 300
D. suzukii Gr63a AMLGAWWFLICEAMSITAHLLAERFQKALKHIGPAAMVADYRVLWLRLSKLTRDTGNAMC 300
D. subpulchrella Gr63a AMLGAWWFLICEAMSITAHLLAERFQKALKHIGPAAMVADYRVLWLRLSKLTRDTGNAMC 300
D. melanogaster Gr63a YTFVFMSLYLFFIITLSIYGLMSQLSEGFGIKDIGLTITALWNIGLLFYICDEAHYASVN 360
D. suzukii Gr63a YTFVFMSLYLFFIITLSIYGLMSQLSEGFGIKDIGLTITALWNIGLLFYICDEAHYASVN 360
D. subpulchrella Gr63a YTFVFMSLYLFFIITLSIYGLMSQLSEGFGIKDIGLTITALWNIGLLFYICDEAHYASVN 360
D. melanogaster Gr63a VRTNFQKKLLMVELNWMNSDAQTEINMFLRATEMNPSTINCGGFFDVNRTLFKGLLTTMV 420
D. suzukii Gr63a VRTNFQKKLLMVELNWMNSDAQTEINMFLRATEMNPSIINCGGFFDVNRSLFKGLLTTMV 420
D. subpulchrella Gr63a VRTNFQKKLLMVELNWMNSDAQTEINMFLRATEMNPSIINCGGFFDVNRSLFKGLLTTMV 420
D. melanogaster Gr63a TYLVVLLQFQISIPTDKGDSEGANNITVVDFVMDSLDNDMSLMGASTLSTTTVGTTLPPP 480
D. suzukii Gr63a TYLVVLLQFQISIPTDKGDSDGATNITVVDFVMDSLDNDMSLMGATTPSTTTAGTTMAPP 480
D. subpulchrella Gr63a TYLVVLLQFQISIPTDKGDPDGATNITVVDFVMDSLDNDMSLMGATTPSTTTAGTTMAPP 480
D. melanogaster Gr63a IMKLKGRKG C-terminus 489
D. suzukii Gr63a IIKQKGRKG C-terminus 489
D. subpulchrella Gr63a IIKQKGRKG C-terminus 489
B
predicted
Spike rate at onset (spikes/s)
500
400
300
200
100
SUB-GAL4 > UAS-SUZ
SUB-GAL4 > UAS-SUB
C
Average spike rate at onset (spikes/s)
CDS
SUZ
SUB
MEL
predicted
***
***
ns
MEL SUB SUZ
*
**
ns
cis-regulatory
Spike Rate (spikes/s)
200 250 300 350
D
Average spike rate at onset (spikes/s)
SUB
MEL
MEL SUB SUZ
*
**
cis-regulatory
E
Average spike rate at onset (spikes/s)
CDS
SUZ
SUB
MEL
***
***
MEL SUZ
