# Supplementary material for: Molecular evolution of CO2-sensing ab1C neurons underlies divergent sensory responses in the Drosophila suzukii species group
Source: PLoS Genet. 2026 Jan 22;22(1):e1012024. doi: 10.1371/journal.pgen.1012024 (PMC12854461; doi:10.1371/journal.pgen.1012024)

**S1 Table.** Raw Ct values for the antennal qPCR for each species. For each species we used three technical replicates for three biological replicates for both *Gr63a* and the endogenous control TBP.


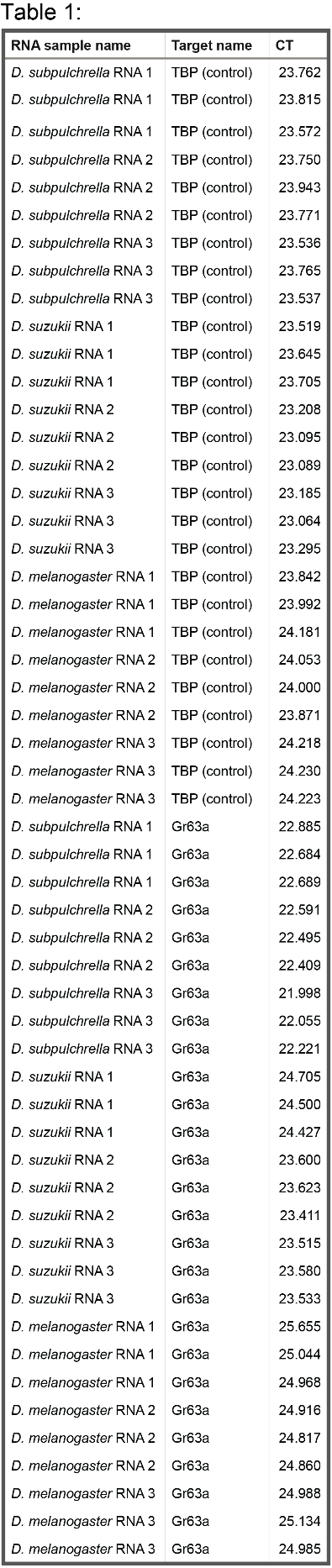

Supplement: S1 Table — For each species, we used three technical replicates for three biological replicates for both Gr63a and the endogenous control TBP. (DOCX) [file pgen.1012024.s005.docx]
