# Supplementary material for: Molecular evolution of CO2-sensing ab1C neurons underlies divergent sensory responses in the Drosophila suzukii species group
Source: PLoS Genet. 2026 Jan 22;22(1):e1012024. doi: 10.1371/journal.pgen.1012024 (PMC12854461; doi:10.1371/journal.pgen.1012024)
Supplement: Attachment — Submitted filename: Gadau_response_to_reviewers_1120.docx [file pgen.1012024.s006.docx]

**Response to editor’s and reviewers’ comments**

**Additional Editor Comments:**

Thank you for submitting your manuscript entitled “Divergent molecular processes drive convergent behavioral innovation in the Drosophila suzukii species complex” for consideration at PLOS Genetics. We appreciate the work that you have accomplished and agree that this manuscript has the broad scientific implications appropriate for the audience of PLOS Genetics. Four experts in the field have evaluated the manuscript and provided feedback. All four reviewers were enthusiastic about the general results but pointed out several points that require additional attention.

Response: Thank you very much for handling our manuscript. We appreciate the positive enthusiasm and comments from the reviewers, as well as their constructive criticism. We have revised the manuscript accordingly.

We request text revisions that temper the causal link between the molecular and neural processes and the behavioral variation studied here to a level commensurate with the results presented in the manuscript.

Response: We have edited extensively throughout the manuscript.

Reviewers brought up several concerns regarding the regulatory differences in the Gr63a that should be addressed, including incorporation of what is known about regulation in other species and a broader comparison of regulatory and coding sequence divergence among additional species of intermediate divergence between the species studied here in the *D. suzukii* complex and *D. melanogaster*. The concern regarding the how the genomic location differences in transgenes and different UAS vectors may confound comparison of expression levels within *D. melanogaster* is of particular importance for the conclusions of this manuscript and would be addressed by the request for quantification of Gr63a transcript abundance.
Response: In addition to the quantitative RT-PCR we performed previously, for this revision, we re-reared the flies and performed quantitative RT-PCR in the transgenic lines. Using these results, we found for the homozygote lines the SSE recording and the quantitative RT-PCR have a Pearson’s correlation of 0.62, supporting that expression of Gr63a genes also play a role in ab1C spike rate. We think this result makes the paper stronger. We thank the reviewer for their valuable comments.

Several reviewers questioned the necessity of the model of single sensillum recordings and suggest that the narrative may be clarified if the model is moved to the supplemental material.
Response: We moved the modeled recordings to the supplemental material.

Justification for experimental conditions and demonstration if the species exhibit the same behaviors at lower, more ecologically relevant levels of CO2.

Response: Added.

The reviewers additionally provided useful comments that would help clarify portions of the manuscript.

Response: We thank all four of the reviewers for their thoughtful comments.

**Reviewers' comments:**

Reviewer's Responses to Questions

**Comments to the Authors:
Please note here if the review is uploaded as an attachment.**

Reviewer #1: Gadau et al. investigate the genetic basis of adaptation by examining how CO2 influences oviposition behavior and sensory neuron activity across three Drosophila species. Using a combination of behavioral, electrophysiological, and genetic approaches, they argue that convergent oviposition traits have evolved through distinct genetic mechanisms—a coding sequence change in one species and a cis-regulatory change in another. The authors developed a refined oviposition assay and demonstrated that CO2 differentially affects both oviposition site preference and egg-laying quantity among species. Single sensillum recordings from CO2-sensitive ab1c neurons expressing GR63a revealed species specific differences in CO2 sensitivity. To dissect the roles of coding versus regulatory changes, the authors generated new transgenic lines that allow independent manipulation of each. These tools showed that a GR63a coding change in one species and a GR63a regulatory change in another species enhance ab1c neuron sensitivity to CO2. Finally, they examined the respective contributions of coding and regulatory changes to oviposition behavior, finding that distinct genetic changes can converge on similar egg-laying suppression traits.
Response: We thank the reviewer for the great summary of our work and the constructive suggestions.

The authors demonstrate a striking cross-species variation in ab1c neurons’ CO2 sensitivity, and the data support the idea that this variation results from convergent evolution of coding and cis-regulatory sequences. However, the relevance of these neuronal changes to behavioral variation remains unclear. The authors show that oviposition behavior differs between *D. melanogaster* and both D. subpulchrela and *D. suzukii* in two key ways: only *D. melanogaster* avoids 1.0% CO2 as an oviposition site, and only *D. melanogaster* suppresses egg-laying in response to 1.0% CO2. As the authors acknowledge in the Results and Discussion, changes in ab1c neuron CO2 sensitivity do not explain differences in either oviposition site preference or egg-laying suppression. Consequently, the genetic basis for the observed behavioral divergence across species remains unresolved. Thus, the current data fall short of supporting the manuscript’s central claim—highlighted in the Title and Abstract—that divergent molecular processes drive convergent behavioral traits. In my view, the most compelling finding of the manuscript is the interspecies differences in ab1c neuron CO2 sensitivity of ab1c neurons and its underlying genetic mechanisms, even though the behavioral data suggest that these neural differences are not directly linked to the behavioral variation studied here.

Response: We agree with the reviewer that the SSE results are the most solid findings. However, this study represents a tremendous amount of work spanning six years, and it was a difficult decision not to highlight all aspects of it in the title. Following your advice, we have revised the title to “Molecular evolution of CO₂-sensing ab1C neurons underlies divergent sensory responses in the *Drosophila suzukii* species group,” and we have rewritten the abstract and extensively edited the manuscript accordingly.

In the transgenic experiments, the authors carefully considered heterozygosity versus homozygosity to ensure fair comparisons. However, two potentially important differences exist between the *D. melanogaster* transgenic lines and those used for D. subpulchrela and *D. suzukii*. First, the Gal4/UAS constructs were inserted at different genomic loci: DmelGr63acis-GAL4 and DmelGr63aCDS-UAS were randomly inserted (Weiss et al., 2011, Neuron; Jones et al., 2007, Nature), whereas the D. subpulchrela and *D. suzukii* constructs were targeted to defined sites (attP2, attP40, or VK00027). Because genomic position can strongly influence transgene expression levels, this difference complicates cross-species comparisons. Second, the UAS vectors differed: DmelGr63aCDS-UAS used the pUAST vector with 5xUAS (Jones et al., 2007, Nature), while the other constructs used pJFRC7 with 20xUAS, likely resulting in different expression levels. These differences make it complicated to determine whether the observed variation in CO2 sensitivity truly reflects differences in coding sequences or cis-regulatory elements.

Response: Thank you for bringing up this concern. We understand that genetic background and the number of UAS sites can affect the intensity of the results, and we do not disagree with this point. However, the actual differences between the Gal4 and UAS lines are not entirely proportional to the number of UAS sites or the genetic background. During the revision, we performed quantitative RT-PCR on the transgenic lines and found that the fold differences ranged from 2 to 6-fold (new S4 Fig). For our transgenic lines the quantitative RT-PCR indeed showed that *D. suzukii* UAS shows a stronger expression than other lines. We also performed immunostaining, although we did not quantify the signals due to the overall uncertainty of quantifying immunostaining signals, strength of different GAL4 lines in driving UAS-GFP differs by a bit, not by orders of magnitude.

In the analysis of single sensillum electrophysiology data, the authors estimated the responses of homozygous lines through extrapolation. However, this approach was validated under only one condition (cis-SUB > CDS-SUB), and it remains unclear whether the predicted spike rate for cis-SUB > CDS-SUZ—which is substantially higher than any observed response—is plausible. Since the same conclusion is supported even without these extrapolated values (Fig. S3B, C), the necessity of including this modeling is unclear.

Response: We understand your concern. We had predicted that cis-SUB > CDS-SUZ would generate the strongest signal, and the fact that it is lethal for unclear reasons, despite many thousands of crosses, leads us to believe this might represent a real effect. It is unfortunate that we were unable to establish a stable strain. Nevertheless, we acknowledge your concern and have removed the bulk of the results and moved the figure into supplementary information.

In the analysis of behavioral data from transgenic lines, it is unclear how the authors addressed heterozygosity versus homozygosity. Are the conclusions consistent when comparisons are limited to homozygous flies? Additionally, it is not explained why obtaining homozygotes was more challenging for the behavioral experiments than for the single sensillum electrophysiology experiments.

Response: The results remain consistent—though the p-values are not as small—when using only the homozygous lines. Regarding your second question, we do not have a clear explanation for why obtaining cis-SUB > CDS-SUZ homozygous lines is more difficult. We spent more than a year performing multiple independent crosses and screened many thousands of flies for this line, but without success. It is very puzzling to us, as one would not expect neural sensing of CO₂ to result in a lethal phenotype, but beyond this, we have no clear explanation. It is baffling that the strain that under our hypothesis would produce the strongest sensitivity to CO₂ is the one that we cannot generate, we think it is worth including in the manuscript to alert future work in this direction.

Line 196: “found that the substantial number of substitutions in the amino acid sequence have evolved at the c-terminus.” Please indicate the C-terminal region in Fig. S3A so that readers can easily confirm this claim. It would also be helpful to discuss the potential functional implications of the selective accumulation of mutations in the C-terminal region.
Response: We thank the reviewer for this suggestion. In the revised version, we have annotated the C-terminal region of the protein in Fig. S3A to make its location clear to readers. We now also added the following information about the c-terminal and N-terminal region in the paragraph starting at line 196-206. “We also compared the *Gr63a* coding sequence between *D. suzukii*, *D. subpulchrella*, and *D. melanogaster*…” .

Fig. 3B, The blue and orange highlights are difficult to see. Most of the sequence appears in pink and red. Also, it is challenging to evaluate from this panel whether the majority of the changes are clustered in the C-terminus.

Response: Thank you for your comments, we have updated the figure. It now shows the full amino acid sequence and structure. The C-terminus is now also more clearly defined.

Line 198: “*D. subpulchrella* and *D. suzukii* only differ 199 from each other at two amino acid sites: sites 48 and 450” These two changes appear to underlie the differential CO2 sensitivity between CDS-SUB and CDS-SUZ. Are both changes located in the C-terminus? This specificity is striking and may deserve further discussion in the text, particularly regarding how such minimal changes could yield substantial functional differences.

Response: Thank you for your comment. We also found this striking and agree that it deserves more discussion. We have added more details in the manuscript under the section “Transgenic *D. melanogaster* flies expressing *D. subpulchrella* and *D. suzukii* Gr63a CDS or cis-regulatory element” (line 184-218) and have added a paragraph in the discussion. What we did not discuss was that there is a slight chance that synonymous mutations also play a role in functional divergence, however, we preferred not to discuss this since we have no evidence in this direction.

Line 249: “However, this finding was not corroborated by the spike rate data, and it may be attributed to the contribution of nearby neurons when calculating LFP.”
Does this imply that the Gal4 driver used for expressing Gr63a may have also driven the expression in neurons adjacent to ab1c, with different CO2 sensitivities between *D. melanogaster* and *D. subpulchrella* CDS? As currently written, it is difficult to understand what mechanism the authors have in mind for the discrepancy between LFP and spike data.

Response: No, the GAL4 drivers used for expressing Gr63a do not drive expression in adjacent neurons. We confirmed this by expressing a GFP reporter under the same driver and observed fluorescence only in the V-glomerulus, where ab1c neurons project. The discrepancy between the LFP and spike rate data reflects the nature of single-sensillum recordings, which are extracellular. In this technique, the electrode is inserted into the sensillum lymph, and each sensillum typically houses 2–4 neurons that share a conductive extracellular space. Because this space is shared, the transmembrane currents from one neuron can alter the extracellular potential around neighboring neurons. Consequently, the local field potential (LFP) represents the summed extracellular currents of all neurons within the sensillum, not the membrane potential of a single neuron.

In contrast, the spike rate data reflect the firing of the specific neuron of interest. The CO₂-responsive neuron (ab1c) can be identified by its distinctive spike amplitude and waveform, allowing us to isolate and track its firing before and after odor onset. Therefore, the spike rate data provide a more specific measure of the CO₂ response than the LFP.

We’ve added to the section so that it may be more informative for readers (line 269-273). It now reads “This difference was not supported by the spike rate data and likely reflects the contribution of nearby neurons to the LFP signal. Because the LFP represents the summed extracellular currents from all neurons within a sensillum rather than the membrane potential of a single neuron [47], variations in neighboring neuronal activity can influence the LFP independently of the CO2-sensitive neuron’s firing. Therefore, LFP and spike rate measurements are not always expected to align.”

Reviewer #2: In their manuscript entitled “Divergent molecular processes drive convergent behavioural innovation in the Drosophila suzukii species complex” Gadau and colleagues describe a differential sensitivity to CO2 between three Drosophilid species (suzukii, melanogaster, and the intermediate-phenotype subpulchrella) as arising through two distinct pathways: alteration of the coding sequence of a receptor (Gr63a) and the evolution of a cis-regulatory element controlling expression of the same receptor. This builds on previous work that identifies the CO2 receptor (Gr63a) as being differentially expressed in subpulchrella and the subject of positive selection in suzukii.

In general the experiments are well controlled and described, the data are clearly presented, and the interpretation of said data is conservative. The implications for the evolution of novel behaviours are clearly interesting and important. I have a few comments below for the authors that might strengthen the arguments they put forward, particularly surrounding the interpretation of qPCR results from wild-type flies of three species then extrapolated to transgenic expression experiments using the Gal4 > UAS system with three different CREs.

Response: Thank you for your positive comments and constructive suggestions. We have spent the past several months addressing your feedback, particularly those related to quantifying the strains used for SSE recordings. Please see the detailed responses below.

Major comments:

- the finding that high levels of CO2 seemed to suppress egg-laying in melanogaster is intriguing, though I struggle with the choice of 1% CO2 in this assay as the authors themselves state that ripe fruit is more typically associated with ~0.16% CO2 (nearly an order of magnitude less). are the flies just generally less active at such high concentrations? is this known? is 1% standard? It seems important to test egg-laying behaviour at lower levels of CO2 to see if the phenotypes observed here are in the realm of possibility for flies experiencing ripe fruit as opposed to piped in gas in an olfactometer at much higher concentrations.

Response: Thank you for this thoughtful comment. We chose 1% CO₂ because previous work has shown that walking *D. melanogaster* avoids CO₂ concentrations between 0.1 and 1% (Suh et al. 2004,<https://doi.org/10.1242/jeb.02297>). Thus, while ripe fruit may contain ~0.16% CO₂, we used 1% because it falls within a range where *D. melanogaster* shows robust behavioral responses, and it ensured that the CO₂ side of our chamber maintained higher CO₂ levels. We were worried that a lower CO₂ concentration may not cover the whole “high CO₂” side in elevated CO₂. At these concentrations flies do not appear to be less active.

Higher CO₂ concentrations (3–5%) have been reported to increase activity and even attraction, particularly in starved flies or during peak activity windows such as dawn and dusk (Wasserman et al. 2013,<https://doi.org/10.5061/dryad.2s8422f>). These effects are mediated through different receptor pathways (e.g., Ir25a) and are more relevant to food-search behaviors. To avoid activating these food-related pathways, we deliberately chose a concentration below 3% CO₂ and did not starve our flies. Instead, we removed oviposition substrate for 24 hours to ensure females were motivated to lay eggs. Under these conditions, gravid *D. melanogaster* females showed suppression of egg laying but no attraction to CO₂.

- The increase in gene expression of Gr63a (as measured by transcript abundance) in subpulchrella could be a result of (at least) two factors: an increase in transcript abundance within the same cells, or expression of 63a in novel cells. The expression pattern showing innervation of the V-glomerulus in all conditions seen in Figure 5 appears to make the second option unlikely, and the lethality observed with the sub-CRE and the suz-CDS appears to suggest that the CRE does in fact drive differential levels of transcript abundance in a transgenic melanogaster context. However, the transcript abundance in the transgenic context is not quantified (e.g. by qPCR) and given that there is the potential for additional amplification from the Gal4 > UAS system, it would be important to see these numbers for the 9 transgenic combinations tested in figures 4 and 5 so that we can compare them to the wild-type abundances observed for the three species.

Response: Thank you for this comment. We spent the past five months generating quantitative RT–PCR results, which required considerable effort. We have now reported these results in line 275-283. Our data support that the enhancer from *D. subpulchrella* indeed drives higher Gr63a CDS expression, suggesting that the findings are consistent with, but more nuanced than, the quantitative RT–PCR results of EGFP. It is worth noting that we were unable to generate homozygous lines for several strains, as these stocks were maintained with balancer lines in the lab. Despite several months of attempts (and previous success with similar efforts), we were unsuccessful this time. Based on this observation, we have revised the text to tone down our statements regarding the lethality of the homozygous strains. We still believe the lethality is associated with the transgenic lines, but we prefer not to draw firm conclusions without stronger supporting evidence.

Minor comments:

- transcript abundance (as measured by qPCR) does not necessarily predict protein abundance, and I would like to see that issue explicitly acknowledged and discussed in the text… in addition, do we know for sure that increased levels of receptor protein complexes leads to increased sensitivity? It seems like an ok assumption, but I’m not aware of any studies making this link explicitly. This all needs to be discussed.

Response: We were unable to directly measure the protein abundance of Gr63a because an antibody is not available. Instead, we used GAL4 to drive EGFP expression and visualized the resulting signal. While this approach is not ideal, it is rigorous compared to methods used in other studies. Given that Gr63a-related GAL4-UAS lines of *D. melanogaster* have been reported in several previous studies, we agree with you that this is an acceptable assumption. We have also conducted a Quantitative RT-PCR with the transgenic lines and found that the trans Gr63a transcript abundance was positively associated with the strength of the SSE responses (Pearson’s R = 0.62, S4B Fig), suggesting that lines with higher expression tended to exhibit more robust neural activity in *D. melanogaster*. This relationship does imply that differences in transgene expression, potentially due to the different enhancer or CDS elements likely accounts for some of the variability observed across SSE recordings. We added the experiment and interpretation to lines 275-283 in the manuscript.

- line 302 misplaced reference 16

Response: Edited.

- Line 201 - UAS is not a gene - this section should be tightened up as creating plasmids is not the same as creating transgenic flies and it is difficult to determine exactly what was done here. The methods section starting line 413 did not clarify exactly where these lines ended up - Attp2? Attp40? Why are multiple landing sites mentioned, and which constructs ended up where?

Response: Thank you for your comment, the section was updated. Please see lines 457-470.

- Line 215 should read ‘then crossed’ - in general the figure legend for this figure is not clearly described.

Response: Thank you for your comment, the figure legend was updated and now reads: GAL4 driver lines carrying each species’ cis-regulatory element were crossed with a Gr63a¹ knockout line and then with one of three UAS lines containing the Gr63a coding sequence (CDS) from each species cloned into a UAS vector. This generated nine transgenic lines in total (highlighted in the green box), representing all combinations of Gr63a cis-regulatory elements driving each species’ Gr63a CDS.

Reviewer #3: A controversial question in 21st century evolutionary developmental biology has been whether regulatory changes or coding sequence changes drive interspecific phenotypic divergence. In this paper, Gadau and colleagues examine a gustatory receptor in two closely related pests (*D. suzukii* and *D. subpulchrella*) and claim that both processes have played a role in the evolution of a novel response to carbon dioxide, with coding sequence changes predominating in *D. suzukii* and regulatory changes in *D. subpulchrella*.

Response: We thank you for your positive comments and helpful suggestions.

While the results are intriguing, I have questions about how the study was conducted and the authors' interpretation of their findings:

1) The authors make no mention of non-pest species that are more closely related to *D. suzukii* and *D. subpulchrella* than the model organism *D. melanogaster*. What is the response of a fly such as *D. biarmipes*, for example, to carbon dioxide? Where does this species prefer to lay its eggs in their oviposition experiments? If the authors have not carried out these experiments, they could at least use the *D. biarmipes* genome to examine how the gustatory receptor they focus on (Gr63a) diverged prior to the evolution of the pest clade. Furthemore, they should mention the importance of examining *D. biarmipes* and other closely related species in the Discussion.

Response: Thank you for bringing up *D. biarmipes*. We have previously (DOI: [10.1093/molbev/msab048](https://doi.org/10.1093/molbev/msab048)) examined *D. biarmipes* in the context of egg-laying on ripe and rotten fruits and found that while it is less likely to oviposit on rotten fruit than *D. melanogaster* it does not exhibit as extreme of a preference switch as *D. suzukii* and *D. subpulchrella*. We also only have an inbred *D. biarmipes* line in the lab, and it is not ideal to conduct behavior experiments with inbred lines. For those reasons we decided to focus on *D. suzukii* and *D. subpulchrella* for our experiments as these have a more extreme egg-laying phenotype. We did not conduct egg-laying experiments in the context of CO₂ with *D. biarmipes*. However, when aligning the 4 species, *D. biarmipes* shares the highest identity with *D. melanogaster* (95.5% versus 95.3% and 95.05% with *D. suzukii* and *D. subpulchrella*, respectively). This suggests that *D. biarmipes* may behave more similarly to *D. melanogaster* in the context of CO2 and egg-laying. In addition, at the three sites that differ between *D. suzukii* and *D. subpulchrella*, *D. biarmipes* shares the same amino acids as *D. melanogaster*, suggesting that they may not share the same sensitivity shift. We’ve added a section in the discussion to address your comment. Please see lines 364-372.

2) I find it interesting (and possibly surprising) that the results for the *D. suzukii* and *D. subpulchrella* Gr63a coding sequence differ dramatically when there are only two amino acid sequence differences (line 199). I believe that examining the *D. biarmipes* sequence could shed light on this issue.

Response: Yes, testing the effects of these amino acid changes will be very important in the future. However, this is technically challenging, as performing single-site genome edits in non-melanogaster species remains difficult, and achieving a homogeneous genetic background adds further complexity. We also examined the sequences in *D. biarmipes* and found that, for all the sites mentioned above, *D. biarmipes* shares the same amino acids as *D. melanogaster* (line 364-372). Thus, *D. biarmipes* likely represents the ancestral state in our experimental context.

3) From the Methods section, it appears that the authors assumed that the regulatory region for Gr63a, in each species, was in the 1 kb upstream of the start codon (lines 417 to 426). This is a dubious assumption, however, and I believe they should discuss what is known in *D. melanogaster* (and possibly other species) about the regulation of this gene. Furthermore, do the regulatory regions they identified contain binding sites for upstream transcription factors? How diverged are these regulatory regions between *D. suzukii* and *D. subpulchrella*?

Response: Thank you for these questions. We used a 2.6 kb upstream region that encompasses the Gr63a regulatory elements (we also generated the 1kb strain following another published study but that were not used for later studies), as this region was sufficient to drive Gr63a expression in *D. melanogaster*, following this work (doi: 10.1073/pnas.0700079104). We used the homologous upstream sequences of Gr63a from *D. suzukii* and *D. subpulchrella* for our experimental design. Our ATAC-seq data also support a relatively conserved peak in the promoter/enhancer region of Gr63a in the three species. The sequence divergence of the regulatory regions between *D. suzukii* and *D. subpulchrella* is about 5%. Due to the fact that it is unknown which TFs primarily bind to these promoter and enhancer regions, it remains unclear which nucleotides are essential for changes in expression, and this is an ongoing topic in the field of gene expression regulation. This was updated in line 456-470.

4) I find these results surprising, given how closely related these two species are. I think the authors should carefully consider if there are any alternative explanations for their findings and mention these in the Discussion.

Response: We agree that this finding is indeed surprising, which is why we were very excited about the results. Given that behaviors related to egg-laying and CO₂ response tend to evolve rapidly, it is not unexpected that the underlying genetic basis might also evolve quickly. While we prefer not to speculate without supporting evidence, if this pattern arose in parallel from the common ancestry of *D. suzukii* and *D. subpulchrella*, it is plausible that the genetic basis evolved independently, with newly derived mutations contributing to similar patterns in each species. However, because we currently lack experimental data for *D. biarmipes*, we cannot definitively answer this question. We appreciate your insightful comment; this question and our response may help readers think more deeply about the evolutionary mechanisms involved.

5) In a few cases (e.g. lines 255 to 262) the authors have including text in the Results that would be more appropriate in the Discussion.
Response: Thanks for the suggestion. We have edited the manuscript to make it more directly related to the results reported, rather than independent discussion.

6) I was confused by the legend to Figure 5. There does not appear to be an explanation for panels A and B.

Response: Thank you for catching this error, we have updated the Figure legend.

Reviewer #4: In this work, Gadau et al. address the genetic and neural basis of behavioral adaptation in *D. suzukii*, a major agricultural pest, and its sister species, *D. subpulchrella*. In parallel with their behavioral shift toward laying eggs in ripe fruits that emit elevated levels of CO₂, the authors establish that both species exhibit a lack of CO₂ avoidance during egg laying compared to *D. melanogaster*. Using single sensillum electrophysiology, the authors demonstrate that in both species, the relevant sensory neurons ab1C, which express the CO₂ receptor Gr63a, are in fact more sensitive to CO₂. Motivated by evidence of positive selection on Gr63a in *D. suzukii* and higher Gr63a expression in *D. subpulchrella*, the authors tested the effects of both protein-coding and cis-regulatory sequences of Gr63a from *D. suzukii*, *D. subpulchrella*, and *D. melanogaster* using chimeric transgenes expressed in a *D. melanogaster* Gr63a null background. They show that the coding sequences of *D. suzukii* and the cis-regulatory sequences of *D. subpulchrella* both enhance CO₂ sensitivity in ab1C neurons. Further behavioral assay testing CO₂ preference during egg laying did not reveal a corresponding behavioral effect. Overall, this work is highly significant: it pinpoints the precise genetic mechanisms in neural evolution for an agriculturally and economically relevant behavioral shift. Notably, while the role of protein-coding changes in sensory receptor evolution is well appreciated, this study provides further novel insights by revealing divergent genetic mechanisms in the same gene drives similar, behaviorally relevant neural changes. The quality of the work is overall excellent, and the transgenic experiments are particularly systematic and rigorous. While the data are strong and broadly support the major conclusions, I have a few suggestions that primarily pertain to the precise interpretation of some results.

Response: We thank the reviewer very much for their positive comments and constructive criticism.

1. The authors have addressed species differences at two levels: the electrophysiological level and the behavioral level (both general avoidance and avoidance in the context of oviposition). The genetic mechanisms underlying evolutionary changes in electrophysiological properties did not produce behavioral effects that align with wild-type phenotypes. This result is well expected. If the ab1C neurons in *D. suzukii* and *D. subpulchrella* exhibited decreased CO₂ sensitivity, a corresponding reduction in behavioral avoidance could be attributed to changes at the peripheral sensory level. However, in this case, since *D. suzukii* and *D. subpulchrella* show increased CO₂ sensitivity, their lack of CO₂ avoidance during egg laying most likely arises from changes at a higher-order processing level that assigns valence to CO₂ signals. Based on this, I have a few suggestions: First, the authors indeed provided a thoughtful discussion and interpretation of their results in the first paragraph of the Discussion section. They may also refine the wording in earlier sections to avoid the impression that increased CO₂ sensitivity is expected to result in reduced CO₂ avoidance, which might confuse readers. Second, while the increased CO₂ sensitivity in *D. suzukii* and *D. subpulchrella* may very well be related to their behavioral adaptation of egg laying on ripe fruit, this connection has not yet been experimentally demonstrated, and the relevant ecological and behavioral context remains unclear (e.g., it is possible that the relevant behavioral phenotypes are in larvae rather than in adults). The authors may tone down their language to avoid potentially premature inference (e.g., Title, Abstract lines 23–24). Finally, the discussion of the contribution of processes “downstream of the peripheral sensory system” (lines 346–348) could be further developed. For example, consider citing Capek et al. 2025 (Nature) and Dürr et al. 2025 (Cell Reports), in addition to Seeholzer et al. 2018. Capek et al. 2025 is particularly relevant to this conceptual discussion.

Response: Thank you for this insightful discussion, we have changed both the title and toned down the language throughout the manuscript. We have added a section in the discussion to further this discussion. Please see lines 394-407 and other sections in the discussion.

2. Protein-coding changes versus cis-regulatory changes: The authors have framed this study within the conceptual framework of evolutionary change through cis-regulatory versus protein-coding sequence alterations in the context of pleiotropic effects. While this framework has been highly influential in studies of phenotypic evolution, I do not think it is fully applicable here. Sensory receptor genes—such as Gr63a, the focus of this study—are typically not pleiotropic in the same sense as developmental master regulators, from which this framework originally derives. Instead, they tend to be uniquely specialized, with restricted expression patterns and dedicated functions. It is in theory possible that cis-regulatory changes might avoid pleiotropic effects if Gr63a is upregulated in specific developmental stage, but this paper has not demonstrated any evidence to suggest that cis-regulatory changes here are less pleiotropic. Additionally, I found the inference that protein-coding changes may have stronger phenotypic effects than cis-regulatory changes (e.g., lines 340–341) not particularly informative, as the relative impact of these changes highly depends on the nature of specific mutations. I suggest removing this interpretation or tune it down. I shall emphasize that regardless, it remains valuable to examine how similar or distinct genetic mechanisms underlie phenotypic evolution. In my view, this is where the primary conceptual contribution of this paper lies: demonstrating that distinct genetic changes within the same gene can drive ecologically relevant convergence of neural electrophysiological traits in two closely related species.

Response: We appreciate the reviewer’s thoughtful feedback regarding the conceptual framework. We agree that the traditional pleiotropy-based argument distinguishing cis-regulatory and protein-coding changes, as applied to broadly acting developmental regulators, may not fully capture the evolutionary dynamics of specialized sensory receptor genes such as Gr63a. Our intent was not to imply that Gr63a experiences the same type of pleiotropic constraints as developmental master regulators, but rather to highlight that both cis-regulatory and protein-coding changes can contribute to evolutionary diversification of sensory neuron function. To respond to your comments, we have revised the framing throughout the manuscript to clarify this distinction. We emphasized the empirical and conceptual contribution of our study: that distinct genetic mechanisms, both regulatory and coding, within the same gene can underlie convergent evolution of neural electrophysiological traits in closely related species.

3. The transgene experiments are slightly confounded by the inability to generate viable homozygous adults of cis-SUB>CDS-SUZ due to lethality. The authors employed a modeling approach to predict the expected homozygous phenotype (spike rate). I don’t think the modeling is necessary for this paper: first, my intuition is that if there are any non-additive interactions between cis-SUB and CDS-SUZ, there is no available “truth” to model from. In another word, this prediction cannot extract information beyond what is already available and therefore does not provide any new or necessary knowledge; second, this predicted result is not critical for the main conclusion (the effect of cis-SUB and CDS-SUZ). Admittedly, I am not an expert in modeling and may not be best positioned to judge its suitability; other reviewers may be in a better position to weigh in. I also found that the result of SUB-GAL4>UAS-SUZ/CyO does not add much, given the differences in gene dosage and the complication of genetic background. I suggest removing these data or moving them to the supplementary data. Relatedly, the lethality of this homozygote is interesting. In the relevant discussion (lines 374–378), it might be worth mentioning that in *D. melanogaster*, Gr63a is also expressed in larvae and may contribute to CO₂ detection (co-expressed with Gr21a)—see Kwon et al. 2007 PNAS.

Response: Thank you for your kind comments on the lines we use. We have moved the modeling results to supplemental data and added quantitative RT-PCR results for the lines used for SSE recordings (line 275-283). We also toned down the results about homozygote lethality as this result is now less of the center of the result (line 356-362).

4. “A stepwise evolutionary system”: For me, “stepwise evolution” implies evolutionary changes occurring in successive steps along a mutational path (e.g., Karageorgi et al. 2020 Nature would be a good example). “Stepwise” might therefore be a confusing term here, given that the mutations (protein-coding and cis-regulatory) suggest independent/parallel (not successive) adaptation.

Response: We did not mean “stepwise evolution” in the same molecular sense as reported by Karageorgi et al. (2020, Nature). Rather, we refer to the stepwise integration of sensory systems described by Karageorgi et al. (2017, Current Biology) and to the stepwise evolutionary process discussed by Durkin et al. (2021, MBE). We cited the relevant papers when mentioning the term in the introduction and removed it from the abstract.

5. Expression level of Gr63a: does the higher expression level of Gr63a revealed by qPCR reflect an increase in gene expression per ORN, or an increase in the number of Gr63a-expressing ORNs (Fig. 3A)? I understand that this may be difficult to quantify directly due to the lack of genetic reagents in *D. suzukii* and *D. subpulchrella*, but perhaps the authors could quantify the fluorescence signal of the transgene mel/suz/sub-cis>GFP? At minimum, the authors should acknowledge both possibilities if they cannot distinguish between them, especially considering that expanding the number of sensory neurons is a common mechanism (e.g., Takagi et al. 2024 Nat Commun as a recent example in Drosophila).

Response: We thank the reviewer for the suggestion. In response to your comments and the comments of other reviewers, we performed quantitative RT-PCR using the transgenic lines we did the SSE recording. The results are reported in line 275-283.

6. When reporting species differences in CO₂ sensitivity of ab1C neurons, the authors should cite Krause Pham and Ray 2015 Scientific Reports, which first demonstrated higher CO₂ sensitivity in *D. suzukii*.

Response: We cited this paper in the previous version already, and now added this reference again when mentioning ablC neurons. Please see lines 98-99 and 106-108.

7. In places, I find the use of transition words (such as “but,” “however,” “surprisingly”) excessive, disrupting the logical flow (e.g., lines 92–96; lines 286–290). I suggest that the authors revisit the usage of these words and evaluate whether an emphasis on contrast or exception—which can be subjective—is truly necessary.

Response: Thank you for your comment, we have updated the manuscript to remove excessive language.

8. Line 301: *D. subpulchrella*, *D. suzukii*, and *D. melanogaster* Gr63a converge on the V-glomerulus. Given the specific meaning of “converge” in evolutionary biology, I suggest avoiding this word (also, genes don’t converge on glomeruli). I also think that this piece of data should appear earlier as a validation that the chosen cis-regulatory sequences work as expected in the transgene experiment.

Response: We agree and have changed the word converge to “are expressed”, which is what we meant.

9. Figure 4B: add statistical results for all pairwise comparisons (can use letters to denote significance) because panel C does not fully reveal this information. Similarly 5A and 5B.

Response: Thank you, we have updated the figure accordingly.

10. A rigorous proof-reading is needed. Here are a few examples:
• Line 57: “significantly highly express” → “significantly highly expressed”
• Line 80: “CO2 - CO₂.”
• Line 84: “showing the first signs of a behavioral shift” → “suggesting a behavioral shift in CO₂ response”?
• Line 82: “like the behavior explained previously” → “consistent with previous reports”?
• Line 96: “avoidanec” → “avoidance.”
• Line 302: citation “16” was placed at the beginning of the sentence.
• Figure 1: panels C and D—make Y axis labels consistent.
• Most places: “ab1c neurons”; some places: “ab1C neurons”—make usage consistent.

Response: All edited. Thank you.
